# Supplementary material for: Decision-Making Regarding On-Farm Culling Methods for Dairy Cows Related to Cow Welfare, Sustainable Beef Production, and Farm Economics
Source: Animals (Basel). 2025 Jun 3;15(11):1651. doi: 10.3390/ani15111651 (PMC12153892; doi:10.3390/ani15111651)
Supplement: Supplementary file 1 [file animals-15-01651-s001.zip › animals-3591752-supplementary.pdf]

Questions at the start of the interview

1. Do you agree in participating in the study (informed consent) and that your provided information will be used anonymously? (when no the interview stops)

Questions for Dairy Farmers

2. Can you tell us about your farm?
3. What are the important reasons for you to cull cattle?
4. What considerations influence this decision?
5. Do you feel that your decision-making on this matter is similar to other dairy farmers?
6. To whom do you sell the cattle (livestock trader or directly to the slaughterhouse)?
7. Do you use the same livestock trader/slaughterhouse for all types of cattle?
8. Are you able to transport cattle from your farm on any day of the week, if desired?
9. Do you know which slaughterhouses your livestock trader sells the cattle to?
10. Do you think it is important for animal welfare that regional slaughterhouses remain operational?
11. Have you made arrangements with your trader regarding which slaughterhouses your cattle are sent to, in terms of animal welfare?
12. To whom do you ask for advice about culling cattle that may not be fit for transport or suitable for human consumption?
13. Is it clear to you when a cow is not fit for transport ?
14. Do you experience problems with cows not fit for transport? (Can you explain? How often?)
15. Are you familiar with the Mobile Euthanasia Unit (MDU)?
16. What are your experiences with it?
17. What do you see as the potential advantages and disadvantages of an MDU?
18. Has the process of culling changed on your farm in recent years?
19. Do you have experience with OFES?
20. How and how often have you experienced OFES on your farm?
21. Do you know what conditions a cattle must meet to be considered for OFES ?
22. What is your opinion on these conditions?
23. What is your opinion on the maximum 72-hour period within which OFES in the Netherlands can still take place after an accident?
24. Do you think it is important to maintain the possibility of OFES ?
25. How do you assess the independence of the PVP when assessing OFES ?
26. How do you experience the procedure of stunning and exsanguination compared to euthanasia?
27. When do you decide to euthanasia instead of sent for slaughter/ OFES?
28. What considerations influence this decision?

29. Can you indicate which signs show that the suffering is unbearable and euthanasia is necessary?
30. Who do you consider the appropriate person to euthanize cattle on your dairy farm?
31. Are there people in your area, other than veterinarians, who euthanize cattle?
32. Do the costs of euthanasia prevent you from calling the PVP?
33. Do you believe this can affect the welfare of the cattle in general?
34. Do you think there is a risk that the sector could get negative attention in the media due to farmers euthanizing animals themselves?
35. Do you expect that fellow farmers experience the issues we discussed in the same way?
36. What factors might make this different?
37. Can you imagine that your colleagues use other solutions?
38. What is your opinion on the use of cattle mortality data by dairy companies and private quality schemes?
39. How does this effects culling decisions?
40. Do you think it is desirable/necessary for measures to be taken to improve the welfare of non-transportable cattle? Can you explain?
41. Do you have any comments or questions, or are there any issues that have not been addressed that you would like to discuss?

Questions for PVP's

1. Can you tell us about your practice and services provided to dairy farmers?
2. What role do you play as a PVP in advising dairy farmers regarding the culling of cattle (treatment, transport to a slaughterhouse, euthanasia)?
3. Can you tell us about any differences between dairy farms regarding the culling of cattle?
4. Is the legislation regarding fitness for transport /slaughter clear enough for you to advise farmers on the appropriate culling modes (treatment, euthanasia, OFES, regular transport to a slaughterhouse, or Mobile Euthanasia Unit (MDU) for a specific animal)?
5. Do you experience differences between dairy farms /farmers regarding the demand for and follow-up on your advice about the culling and euthanasia of cattle? Please elaborate.
6. Do you have the impression that animal welfare of cattle is compromised when farmers hold these animals while waiting for the withdrawal time for meat to allow for transport to a slaughterhouse?

Emergency Slaughter

7. How many adult cattle are offered to you for OFES each year?
8. What are common reasons for OFES ?
9. Do you ever experience pressure from farmers during the AM inspection of emergency slaughters?
10. How do you handle this pressure?

11. Do you believe practicing veterinarians can work independently enough to perform official tasks such as assessing OFES?
12. What value do you attach to the principle of OFES in the context of animal welfare?
13. How do you find carrying out the procedures of stunning and exsanguination?
14. Do you see differences between colleagues in terms of the services provided regarding emergency slaughters?
15. How do you experience the collaboration with the competent authority regarding the assessment of OFES?
16. Which euthanasia method do you find the most animal-friendly: euthanasia or stunning and exsanguination?

Mobile Euthanasia Unit

17. Are you familiar with the Mobile Euthanasia Unit (MDU)?
18. Is the Mobile Euthanasia Unit used in the area where you work?
19. To what extent do you assess the Mobile Euthanasia Unit (MDU) as a solution for safeguarding the welfare of cattle with reduced economic value?
20. Do you see any reasons to differentiate between the performance of AM inspections for OFES (pvp) and AM inspections for cattle offered for MDU (official veterinarian)?

Euthanasia

21. Can you indicate how many cattle you euthanize per year?
22. Can you express the number of euthanasia cases in your practice annually as a percentage of the total number of milk-type cattle aged ≥2 years within your practice?
23. Can you indicate what percentage of the cattle you euthanized had a reasonable chance of recovery if treated (but the farmer chose euthanasia over treatment for cost or other reasons)?
24. What factors contribute to a farmer's decision to choose euthanasia over treatment in these situations?
25. Can you indicate what percentage of the cattle you euthanized could have been declared suitable for human consumption after slaughter, based on your expectations?
26. What factors contribute to a farmer's decision to choose euthanasia over slaughter in these situations?
27. In what percentage of euthanasia requests do you believe the farmer has delayed the request to the point where animal welfare has already been compromised?
28. Have you ever refused a request for euthanasia of a cattle?
29. What were the reasons behind this decision?
30. Do you have the impression that cattle with no economic value are killed by farmers themselves or other professions to save on euthanasia costs by the pvp?
31. What is your opinion on this practice?

32. What is your opinion on the use of cattle mortality data by dairy companies and private quality schemes?
33. How does this impact the welfare of cattle?
34. Do you believe additional measures or adjustments in legislation are needed to safeguard the welfare of cattle with reduced economic value?
35. Do you have any comments or questions, or are there any issues that have not been addressed but you would like to discuss?

Questions for Slaughterhouse Operator

1. Are you, as a slaughterhouse operator, asked for advice by dairy farmers, veterinarians, or traders regarding the possibilities for animal transportation (live or for OFES)?
2. How do you assess the quality of the advice given by other professions (livestock traders and veterinarians) to farmers regarding the suitability of an animal for transport and the advice on whether the meat of an animal is suitable for human consumption?
3. Is the legislation regarding transport and OFES clear enough for you?
4. In what way do you think the legislation surrounding transport and OFES impacts the welfare of cattle?
5. Can you indicate how many OFES you process annually?
6. Can you indicate how the number of OFES you process has changed over the years?
7. Could you explain the role of livestock traders in the transport of OFES and the transport of cattle to your slaughterhouse?
8. Do you receive OFES on weekends or in the evenings?
9. Can you explain how the costs for slaughter and OFES are structured?
10. How do you assess the maximum time of 72 hours between an accident and slaughter in relation to animal welfare?
11. What are the most common conditions of cattle that you receive for OFES?
12. Are there specific conditions that often lead to rejection?
13. How do you assess the legislation regarding emergency slaughters?
14. Have you ever had indications or suspicions that the welfare of cattle while alive has been compromised? (e.g., multiple gunshot wounds during OFES)
15. How do you assess the conditions set for the removal of cattle for OFES?
16. Can you indicate in what way the possibilities for OFES influence the welfare of cattle?
17. What limiting factors play a role in applying OFES or transport of cattle to the slaughterhouse?
18. Do you believe the AM and PM inspections are sufficiently uniformly carried out by the competent authority?
19. Do you have the impression that the result of the AM and PM inspection generally achieves its goal (approving meat that is suitable for human consumption and downgrading meat that is not)?
20. What percentage of cattle brought in for OFES is deemed unsuitable during the PM inspection because the official veterinarian judges that the animal does not meet the requirements for emergency slaughter as exceeding max timespan between accident and killing?
21. What percentage of cattle brought in for OFES is deemed unsuitable during the PM inspection because the official veterinarian decides that the meat is not suitable for human consumption?
22. Are the PM inspection decisions for OFES comparable to the inspection decisions for the group of cattle brought in alive?

23. How do you assess the consistency of the evaluations made by the competent authority regarding transport suitability, emergency slaughter, and ante- and post-mortem inspections?
24. Who do you consider the appropriate person to perform AM inspections for emergency slaughters and cattle brought in via MDU (practicing veterinarian or official veterinarian)?
25. How do you assess the professionalism of veterinarians when carrying out the stunning and exsanguination procedures?
26. Have you ever performed this procedure at the request of the farmer/veterinarian?
27. How do you assess the independence of the practicing veterinarian when assessing OFES?
28. Do you see reasons to differentiate between the performance of AM inspections for OFES (practicing veterinarian) and AM inspections for cattle offered for MDU (official veterinarian)?
29. Can you explain in what way slaughterhouses can contribute to preventing welfare issues?
30. Do you believe that the possibility of using a mobile euthanasia unit is valuable/important in terms of animal welfare?
31. How do you assess the role of the livestock trader in relation to the welfare of cattle?

Questions for Livestock Traders

General Information

1.Can you tell us something about your company?

2. Has the way you operate changed in recent years?

3. What are important developments you have observed?

Decision-Making / Advising
4. What are important reasons for farmers to cull cattle?
5. What role do you play in advising farmers on the disposal of cattle?
6. Do you see differences between cattle farms in the type of animals offered that are questionable / unfit for transport / questionable for consumption?
7. In your opinion, is the transport regulation clear?
8. Do you experience problems with cattle that are unfit for transport?
9. Could you elaborate on that?
10. Do you see significant differences between dairy farms in how they make decisions about culling?
11. Do you have the impression that animal welfare is compromised because farmers keep couws during the withdrawal period in order to still send them to a slaughterhouse?
12. Do you see differences in the advice given to farmers by other professionals (e.g.

slaughterhouse operators, veterinarians) regarding cattle disposal?
13. Are there farmers in your client base who impose specific conditions regarding the destination of culled cattle?
14. Can you describe how cattle are traded and how the destination is determined?
15. What influence do you think transport distances have on cattle welfare?
16. In your view, how does legislation concerning transport and OFES affect cattle welfare?
17. Do you ever experience pressure from farmers regarding the transport of cattle for slaughter or OFES ?
18. What causes this pressure?
19. How do you deal with it?
20. How do you assess the consistency of the competent authority's assessments regarding fitness for transport, OFES and ante- and post-mortem inspections?
21. Do you think it is important for regional slaughterhouses to remain available for the sake of animal welfare?
22. Can you explain how slaughterhouses can contribute to preventing animal welfare issues?

Mobile Killing Unit (MDU)
23. Are you familiar with the Mobile Killing Unit (MDU)?
24. What are your experiences with it?
25. In your opinion, what are the possible advantages and disadvantages of an MDU?
26. Do you see reasons to distinguish between ante-mortem inspections for emergency slaughter (done by practicing vets) and those for cattle sent to an MDU (done by official vets)?

OFES
27. Can you describe the services you provide in relation to OFES ?
28. Approximately how many OFES are you involved in per year?
29. What limiting factors play a role in performing OFES or transporting dairy cattle to the slaughterhouse?
30. Is the regulation concerning OFES clear enough in your opinion?
31. What is your opinion on the conditions regarding OFES ?
32. What is your opinion on the 72-hour maximum period after an incident in which OFES is still allowed in the Netherlands?
33. What value do you assign to the concept of OFES in the context of animal welfare?
34. Do you think it is important to keep the option of OFES available for cattle?
35. How do farmers perceive the act of stunning and exsanguination compared to euthanasia?
36. How do you assess the skill of veterinarians in carrying out the procedures of stunning and exsanguination?
37. Do you ever perform these procedures yourself at the request of the dairy farmer or veterinarian?
38. How do you assess the independence of the practicing veterinarian when evaluating OFES ?

Euthanasia

39. When do you advise dairy farmers to euthanize cattle rather than send them for slaughter?
40. What percentage of euthanized, unfit-for-transport cattle do you think would technically have been suitable for human consumption after slaughter?
41. Who do you believe is the most appropriate person to perform on-farm euthanasia of cattle?
42. Do you observe that the cost of euthanasia deters farmers from calling a veterinarian?
43. Do you think animal welfare is compromised because of this?
44. Do you think there is a risk that the sector could receive negative media attention due to farmers killing animals themselves?
45. What is your opinion on the use of cattle mortality statistics by dairy companies and private quality assurance schemes?
46. Do you think measures are desirable or necessary to improve the welfare of cattle that are unfit for transport? Could you explain?
47. Do you have any further comments, questions, or topics that were not addressed but that you would like to discuss?
